# Neurodegeneration and Inflammation—An Interesting Interplay in Parkinson’s Disease

**DOI:** 10.3390/ijms21228421

**Published:** 2020-11-10

**Authors:** Chrysoula Marogianni, Maria Sokratous, Efthimios Dardiotis, Georgios M. Hadjigeorgiou, Dimitrios Bogdanos, Georgia Xiromerisiou

**Affiliations:** 1 Department of Neurology, University Hospital of Larissa, Faculty of Medicine, School of Health Sciences, University of Thessaly, 41110 Larissa, Greece; c.marogianni@gmail.com (C.M.); marysokra@gmail.com (M.S.); edar@med.uth.gr (E.D.); 2 Department of Neurology, Medical School, University of Cyprus, 1678 Nicosia, Cyprus; hadjigeorgiou.georgios@ucy.ac.cy; 3 Department of Internal Medicine, University Hospital of Larissa, Faculty of Medicine, School of Health Sciences, University of Thessaly, 41110 Larissa, Greece; bogdanos@uth.gr

**Keywords:** Parkinson’s, neuroinflammation, neurodegeneration, autoimmunity

## Abstract

Parkinson’s disease (PD) is a neurodegenerative disorder, caused by, so far, unknown pathogenetic mechanisms. There is no doubt that pro-inflammatory immune-mediated mechanisms are pivotal to the pathogenicity and progression of the disease. In this review, we highlight the binary role of microglia activation in the pathophysiology of the disorder, both neuroprotective and neuromodulatory. We present how the expression of several cytokines implicated in dopaminergic neurons (DA) degeneration could be used as biomarkers for PD. Viral infections have been studied and correlated to the disease progression, usually operating as trigger factors for the inflammatory process. The gut–brain axis and the possible contribution of the peripheral bowel inflammation to neuronal death, mainly dopaminergic neurons, seems to be a main contributor of brain neuroinflammation. The role of the immune system has also been analyzed implicating a-synuclein in the activation of innate and adaptive immunity. We also discuss therapeutic approaches concerning PD and neuroinflammation, which have been studied in experimental and in vitro models and data stemming from epidemiological studies.

## 1. Introduction

Parkinson’s disease (PD) is a common neurodegenerative disorder, only second to Alzheimer’s disease [1]. The increased incidence of the disease is related to the aging of the population [2]. Parkinson’s is a multi-systemic alpha-synucleinopathy, which leads to the death of dopaminergic neurons (DA) in the midbrain. Apart from old age, which is a well-known risk factor of the disease, environmental factors and genetic deficiencies also contribute to the degeneration of DA neurons [3]. It is worth mentioning that organic chemicals and pesticides such as 1-methyl-4-phenyl-1,2,3,6-tetrahydropyridine (MPTP) and rotenone, apart from directly harming the DA neurons, cause a long-lasting damage to this specific part of the central nervous system (CNS), affecting DNA methylation and promoting further inflammatory reactions to the brain parenchyma [4,5]. Based on the aforementioned data on environmental factors and inflammation, scientific research has focused on clarifying the possible role of inflammatory mediators in the pathophysiology and the progression of PD.

The acquired knowledge so far, concerning the pathophysiology of Parkinson’s, is limited and at times controversial. Upon data collection from studies conducted in the general field of neurodegenerative diseases and mostly in Alzheimer’s, neuroscientists have discovered an association between degeneration of the brain and sustained inflammation [6,7]. As far as Parkinson’s disease is concerned, the first pathophysiological clues were found in pathological studies of PD brains. The progressive degeneration of DA neurons was identified in substantia nigra pars compacta (SNpc), along with the inclusions of aggregated misfolded a-synuclein, also known as Lewy bodies [8,9]. Further research revealed that these inclusions were also present in the peripheral nervous system, which was elementary to the establishment of the hypothesis that Lewy pathology starts in the periphery and then spreads to the CNS, as classified in the Braak’s staging system [10,11]. In these pathological studies and in more recent animal studies (rat PD models), the inflammatory process was identified in the form of induced activated microglia producing cytokines with the increase in autoreactive T lymphocytes in the periphery and in the plethora of antigen presenting cells and MHC II complexes in the peripheral and central nervous system [12]. Possible mechanisms of molecular mimicry have linked DA neuron degeneration and various virus infections [13,14]. Moreover, a-synuclein aggregations found in the brain and in the gut seem to establish a definite relation between these two seemingly unrelated organs, and inflammation in the gut functions as a possible starting point for neurodegeneration in SNpc [15].

## 2. Inflammation in Parkinson’s Disease (PD)

Inflammation seems to be part of the pathophysiological pathway of Parkinson’s, but we have not yet been able to clarify whether it plays a crucial role in the pathogenicity of the disease or deteriorates and accelerates its progress. Important discoveries about the HLA complex were made in genetic research of the disease [16]. Genome wide association studies have revealed elevated expression in specific genes coding HLA, and at the same time decreased expression of some other HLA types [16]. Similar results were found concerning autoimmune diseases such as inflammatory bowel disease (IBD) and Crohn’s disease [17]. In animal models of PD such as mice and monkeys treated with 1-methyl-4-phenyl-1,2,3,6-tetrahydropyridine (MPTP) and 6-hydroxydopamine (6-OHDA) rats, scientists have tried to replicate the damage caused to the nigrostriatal DA pathway observed in parkinsonian patients [18]. Compared to the human disease, animal models present some inconsistencies, concerning the topology of the degeneration and in some cases, the lack of Lewy body inclusions, but they offer a wide range of information about the inflammation in the CNS [19]. Paraquat and rotenone are used in pesticide/herbicide animal models, which exhibit reduced motor activity and a dose-dependent loss of midbrain SNpc neurons. The latter animal models are usually used to test neuroprotective compounds. The genetic models of PD, on the other hand, are usually transgenic mice that carry mutations in common genes related to PD. They are usually used to study the role of the gene mutations in PD [19]. Some of the results in these animal models confirmed the microglia activation, seen in the postmortem PD brains and the upregulation of pro-inflammatory mediators such as cytokines. The increased level of the peripheral cytokines that act on the endothelial cells of the blood brain barrier (BBB) cause an increase in vascular permeability [20]. The CNS is no longer considered an immunologically privileged site, and the breakdown of the BBB happens during acute or chronic inflammation. These inflamed CNS endothelial cells upregulate the expression of certain adhesion molecules that recruit circulating T cells, monocytes, and attract across the barrier more immune cells and antibodies [20]. The past few years, an effort has been made to fully assess these inflammatory factors, either in the peripheral blood or the CSF of PD patients, and study their alterations in comparison to healthy individuals [21].

### 2.1. Microglia Activation

Microglia represent the phagocytes of the brain and exert both neuroprotective and neurotoxic functions. In a healthy brain, resting microglia surveil the CNS for potential threats, trying to maintain the homeostasis, secreting neurotrophic factors such as nerve growth factor (NGF) and basic fibroblast growth factor (bFGF). A wide range of antigens can activate microglia, ranging from infectious agents, foreign pathogens, prions, or other pathologically-modified CNS proteins, aggregates, and apoptotic cells. Other common stimuli for microglia activation, both in vitro and in vivo, are interferon (IFN)-γ, β-amyloid (Aβ), lipopolysaccharide (LPS), and α-synuclein [22].

The activated microglia participate in neuroinflammation in PD, as indicated from various studies in tissue culture and in animal models of the disease [23]. In postmortem PD brains, significant microglial activation has been found, indicated by an abnormal over-expression of HLA-DR, an MHC-II cell surface receptor, in the affected regions of the brain (mainly in SNpc) [24]. These multiple HLA molecules are expressed by DA neurons and present the digested antigenic peptides on their surface, in order to be recognized by CD4+ T lymphocytes. Although neurons usually lack MHC expression, SN and Locus Coeruleus (LC) neurons have been found expressing MHC after IFNγ stimulation [25]. The previous finding, along with the BBB permeability allowing the infiltration of SN with CD4+ and CD8+ T cells, could explain the pathological findings in PD brains [24]. At the same time, activated microglia secrete a wide range of inflammatory mediators such as TNFα, IL-6, NOS2, COX2, and ROS. These molecules mediate efficient presentation of neoantigens to CD4+ T cells via the MHC-II pathway, leading to cell proliferation, subsequent slow degeneration, and finally the death of DA neurons [26]. MHC-II expressing microglia and CD4+, CD8+ T cells were documented in the SNpc of rat models of PD [27]. The same results have been obtained by positron emission tomographic (PET) analysis in living PD patients [28,29]. Therefore, the chronic microglia activation in PD could exacerbate the condition by producing an excessive amount of these pro-inflammatory and cytotoxic factors [26]. This specific increase in the aforementioned inflammatory factors could be used as a potential biomarker for the early diagnosis and detection of PD progression. On the other hand, the neuroprotective role for microglia was confirmed in a recent study on mice where the microglia’s major role in the clearance of neuron-released α-synuclein was identified [30].

### 2.2. Specific Cytokine Signaling in Parkinson’s Disease (PD)

The expression of cytokines IL-1α, IL2, IL-1β, TNF-α, IL-6, TGF-β, and IFNγ has been implicated in the degeneration of DA neurons in SNpc, following the activation of microglia [31]. The increased levels of pro-inflammatory cytokines found are indicative of an immune system response to the damage of DA neurons. Studies conducted examined the CSF and peripheral blood of PD patients, mainly showing elevated serum IL-1β and IL-6 and elevated TGF-β in CSF [32]. Furthermore, it was found that the mRNA expression of IL-6 was significantly increased in the hippocampus of PD patients also suffering from dementia [33]. Moreover, in the case of TNF-α, it has been found that the inhibition of soluble TNF signaling, with the administration of recombinant dominant-negative TNF inhibitor XENP345, leads to approximately 50% rescue of DA neurons in several animal model experiments [34].

Another important cytokine that has been implicated in the pathogenesis of PD is IL9. IL9 is a pleiotropic cytokine with pro-inflammatory and regulatory functions, depending on the context in which it is induced and the nature of producing cells. IL9 influences the activity of different cell lines in both the immune and central nervous system (CNS). Notably, Th9 cells/IL9 signaling has been associated with neurodegeneration and autoimmune CNS diseases [35]. The important difference compared to other cytokines is the neuroprotective role and support in repair functions that has been attributed to IL9 [36]. Lower IL9 serum concentrations in PD patients have been found recently and might suggest a dysregulation in IL9 signaling that contributes to impaired neuroprotection capacity in PD [37].

Therefore, it seems that there is a pattern of systemic inflammatory markers in patients with PD (i.e., lower levels of IL9 and higher concentrations of CRP, MIP-1β, and TNF-α) that highlights the existence of a specific inflammatory signature [38,39]. In addition, the levels of these markers correlate with the clinical stage of the disease, proving the contribution of peripheral inflammation in PD progression [32,40].

### 2.3. Viral Infections

A possible association between PD and viral infections has been investigated since the first decades of the twentieth century, after the outbreak of encephalitic lethargica [41]. Encephalitic lethargica and postencephalitic parkinsonism occurred several years after the influenza A virus pandemic. Although these two disorders initially were not directly linked to PD, some common pathological features were found in postmortem brain tissue, suggesting a causative effect [42]. These included neurofibrillary tangles and loss of DA neurons in substantia nigra, whereas no Lewy body inclusions were detected.

Similar pathological findings were recognized in animal models infected with the H5N1 influenza virus [43]. Specifically, spreading of the H5N1 from the peripheral nervous system to the CNS caused the activation of certain immune responses in the brain, which led to DA neuron degeneration. Although the dopaminergic loss was transient, the inflammation persisted for a significantly longer period.

In another animal model with the H1N1 virus, which is not a neurotropic virus in mice, an inflammatory process initiates in the periphery, then spreads to CNS and causes neurodegeneration and protein aggregation [44]. Another study that examined H1N1-infected mice, injected with MPTP, found even more elevated levels of microglia activation and consequently degeneration [45]. Additionally, this study has revealed that when a H1N1 vaccine or an antiviral treatment was administered, the inflammation was confined to the damage caused by the parkinsonian drug MPTP and the impact from H1N1 was mostly alleviated.

Immune stimulation from herpes simplex virus-1 (HSV-1) in PD patients, and the effect of a previous infection on the course of PD, have been investigated over the past decade [46,47]. Specifically, studies revealed that HSV1 derived antigens seem to trigger the activation of homologous T-cells and B-cells, that also recognize a-synuclein derived antigens. Findings indicate a possible mechanism of molecular mimicry between HSV-1 and a-syn in the membranes of DA neurons of the SNpc [13,14]. A study on humoral cross-reactivity between a-synuclein and HSV-1 epitope showed that PD patients had higher levels of antibodies against HSV-1 peptides compared to healthy individuals. It also showed the same tendency against human a-synuclein peptides homologous to viral epitopes [14].

Similarly, Epstein–Barr virus (EBV) infection was studied in correlation with PD. Data from epidemiological studies showed that seropositivity in EBV was higher in parkinsonian patients than in the general population [48]. Additionally, molecular mimicry mechanisms were observed, since it was found that antibodies directed against the latent membrane protein 1 (LMP1) of EBV, present in genetically predisposed individuals, cross-react with the homologous epitope on a-synuclein, inducing its oligomerization [49]. Neurohistological studies revealed the presence of immunoglobulins near DA neurons in the brain tissue of PD patients. This finding probably indicates an interaction between microglia and B lymphocytes [14].

### 2.4. Autoimmunity and the Role of A-Synuclein

A-synuclein aggregates are found mainly in the SNpc in PD, but they can also be found in neurons throughout the CNS, the peripheral nervous system (PNS), sympathetic ganglia, and the myenteric plexus of the intestines [50]. In addition, a mutation in the SNCA gene, which produces α-synuclein, is responsible for the monogenetic forms of PD [51]. The most highly organized a-synuclein aggregates are Lewy bodies, which are also composed of ubiquitin and many other cellular proteins.

It is known that the phosphorylation, misfolding, and abnormal accumulation of a-synuclein plays a crucial role in the pathophysiology of PD. Activated microglia proceed to the phagocytosis of a-synuclein aggregates, inducing an immune response, which ultimately leads to neurodegeneration [52]. Many animal and in vitro studies have clearly shown that a-synuclein acts as a potent inflammatory stimulator of microglia [53]. Specifically, after the injection of short α-synuclein fibrils, chemokines were produced and major histocompatibility complex II (MHCII) induction in microglia was promoted, along with peripheral macrophage and monocyte recruitment. MHCII induction persists over time and even seems to spread to other brain territories (as the striatal) six months after the injection. The microglia activation and the subsequent immune response spread across the brain along with the a-synuclein inclusions, ultimately leading to dopaminergic neurodegeneration. This process highlights the implication of the innate immune system to the disease course, and mostly the fact that α-synuclein fibrils are potentially involved in the early stages of PD, and therefore they are promising candidate biomarkers for the preclinical PD [53,54].

Another promising feature in the preclinical and early stages of PD, according to a recently published case-control study, are a-synuclein-specific T cell responses [55]. These T cells were detected prior to the diagnosis of motor PD and declined thereafter. The specific T cell reactivity to α-syn-derived epitopes indicates autoimmune features in PD. Except for the correlation with the time of diagnosis, this specific T cell reactivity was also correlated with age and a low levodopa equivalent dose (LED < 1000 mg/day) [55].

Another study measured the levels of CSF a-synuclein using ELISA and found that mean a-syn concentrations were significantly lower in PD, MSA, and Lewy Body Dementia patients compared to other neurological patients. Unfortunately, this method had low specificity, but had high positive predictive value that could be used for stratification of patients in future clinical trials [56].

A novel method for the detection of abnormal a-synuclein is real-time quaking-induced conversion (RT-QuIC) [57]. This method uses aggregated a-synuclein in order to cause further aggregation of non-aggregated a-synuclein in a cyclic manner. Levels of abnormal CSF a-synuclein were detected with RT-QuIC with a sensitivity of 95% and a specificity of 100% [57].

A possible role of the adaptive immune system has also been implicated in the pathogenesis of neurodegeneration in PD. Experiments in human and animal models have shown that both CD8+ and CD4+ T cells infiltrate the substantia nigra of PD patients [58,59]. More specifically, experimental data from a mouse model suggest a PD-associated shift to a Tc1/Th1- type immune response, based on the increased ratio of CD8+ Tc to CD4+ Th and the increased ratio of IFN-γ producing T cells to IL-4 producing T cells [60]. This imbalance toward pro-inflammatory Th cells (mainly Th1) than anti-inflammatory cells (Th2, Treg) is a likely contributing factor to the sustained neuroinflammation leading to neuronal degeneration. Accordingly, a case-control study was designed to address whether PD is associated with T cell recognition of a-syn epitopes presented by a specific MHC allele [61]. Sultzer et al. featured a T cell response largely mediated by IL-4 or IFNγ-producing CD4+ T cells, with potential contributions from CD8+/IFNγ producing T cells. They also discovered that T cells respond to α-syn epitopes found in both extracellular native a-syn and fibrilized a-syn.

As far as the B cell involvement is concerned, findings are controversial. Few studies have shown a decrease in the population of B lymphocytes and others have detected no alteration in the peripheral blood of PD patients [62,63]. Findings from a recent study focused on naturally occurring antibodies that target Parkinson disease pathology [64]. The researchers isolated memory B cells, producing anti-a-synuclein antibodies, and discovered that three of these antibodies inhibited the seeding of intracellular a-synuclein aggregation. In this case, IgGs had a protecting role in PD pathogenesis.

### 2.5. Gut–Brain Axis in PD

The presence of Lewy body formations in the gut has led the scientific community to further investigate this possible gut–brain connection in PD patients [11]. Environmental factors such as gut microbiome probably function as a trigger factor for the aggregation of a-synuclein [65]. Animal studies in transgenic mice confirmed the above hypothesis. These studies showed that overexpression of human a-synuclein in mice leads to the development of parkinsonian symptoms along with aggregations of a-synuclein in the gut and brain [66]. In contrast, when these mice are bred in germ free conditions or are treated with broad spectrum antibiotics, they show no indication of parkinsonism or consistent pathological signs in the brain [66].

Given the possible interplay between the gut microbiota and microglia, the composition of intestinal bacteria may affect the disease pathogenesis and may even be associated with specific clinical parameters [67]. In a recent study, it was found that the relative abundance of Enterobacteriaceae was positively associated with the severity of postural instability and gait difficulty [67].

Studies on PD patients showed persisting bowel inflammation in comparison to healthy controls. Originally, the effects of inflammation on the enteric nervous plexus were found to stimulate the CNS via the vagus nerve [68]. In fact, there are cohort studies of vagotomized patients that examined whether the vagotomy was associated with the risk of PD. Both studies found a potential decreased risk for PD after truncal vagotomy compared to patients who underwent superselective vagotomy and the general population [69,70].

The compromised BBB seemed to help the penetration of the peripheral proinflammatory factors and other activated immune cells to the brain parenchyma. More specifically, scientists analyzed the mRNA expression profile of pro-inflammatory factors such as cytokines and glial markers in tissue specimens from colon biopsies of PD patients and controls [71]. They found significant increase of TNF-α, IFN-γ, IL-6, and IL-1β in parkinsonians compared to healthy individuals. It seems that all these activated immune cells contribute to the BBB disruption and neuroinflammation. Similar findings were identified in stool specimens, where elevated proteins related to angiogenesis were recognized and elevated cytokines such as IL-1α, IL-1β, and IL-8 were identified in PD patients [72].

Another recent study in mice where their duodenal intestinal lining had been inoculated with a-synuclein preformed fibrils (PFFs) showed that PFFs directly disrupt ENS connectivity and allow a-synuclein histopathology to progress to the brainstem [73]. Most importantly, this seemed to happen in an age-dependent manner, with the older mice having reduced levels of striatal dopamine.

## 3. PD-Associated Genes and Neuroinflammation

Many recent studies indicate a special connection between some PD-associated genes and the immune response of microglia and astrocytes of the CNS [74]. The involved genes are the *α-synuclein gene (SNCA), leucine-rich repeat kinase 2 (LRRK2) gene, PTEN-induced putative kinase1 (PINK1) gene, Parkin, and DJ-1 gene* [75].

As above-mentioned, SNCA missense mutations lead to the accumulation of a-synuclein and activation of microglia, neuroinflammation, and degeneration of the striatal neurons [51].

*LRRK2* gene variations function as risk factors for both familial and sporadic PD. Moreover, *LRRK2* has been detected in B lymphocytes and macrophages, indicating an active participation in immune response [76]. Many studies present interesting results concerning the expression of *LRRK2* in primary microglial cells of adult mice and the secretion of proinflammatory cytokines from these activated microglia after inflammatory stimuli such as lipopolysaccharide (LPS) [77]. On the other hand, LPS-stimulated *LRRK2* knockdown reduces the secretion of TNF-α and the production of iNOS, and decreases the activation of NF-κΒ transcriptional activity in microglia. LPS-stimulated *LRRK2 (R1441G)* transgenic microglia have been found to induce cell death when added to neuronal cultures [78]. The role of *LRRK2* in periphery inflammation was revealed after IFN-γ treatment, where it was found that the expression levels of *LRRK2* were significantly raised, a fact that indicates that LRRK2 transcription is tightly regulated in the physiologic condition [79]. These data indicate that patients carrying *LRRK2* mutations present enhanced neuroinflammation and therefore excess neurodegeneration and progression of the disease. *LRRK2* gene variants have been also associated, apart from PD, with Crohn’s disease (CD) [80]. CD is a form of inflammatory bowel disease with a higher prevalence in Ashkenazi Jewish populations. A recent genetic study found a newly identified *LRRK2* variant, *N2081D*, which is associated with a higher risk of CD and an increased risk of PD [81]. They have also found a *LRRK2* variant with protective features, which lowered the risks for both diseases.

Missense mutations in the *PTEN-induced putative kinase1 (PINK1)* gene are the cause for an early-onset form of Parkinson’s that is recessively inherited [82]. The main aberration elicited by *PINK1* mutations are mitochondrial damage, as *PINK1* directly phosphorylates parkin, enhancing its activity [83]. Studies have shown that *PINK1* deficient mice produce higher striatal levels of IL-1β, IL-12, IL-10, and TNF-α following systemic LPS treatment [84].

*Parkin* mutations are the most common cause of recessively inherited PD. Normally, this gene encodes an E3-ubiquitin ligase [85]. Findings in *Parkin* knockout mice showed excessive degeneration of DA neurons after LPS treatment and increased neuronal vulnerability to rotenone toxicity. Specifically, it was found that *Parkin*-null microglia after LPS treatment produced higher levels of pro-inflammatory cytokines such as TNF-α, IL-6, and iNOS [86].

Another PD-associated gene that has been studied in order to investigate its possible connection with inflammation is *DJ-1*, a gene that is principally expressed in astrocytes and microglia. Thereby, *DJ-1* knockout mice were studied, and higher levels of cyclooxygenase-2 (COX2) and IL-6 were found in astrocytes after LPS treatment [87].

Consequently, there are variants of the aforementioned genes that tend to produce pro-inflammatory cytokines after treatment with LPS, but it still remains elusive as to how exactly these changes affect the course of disease.

## 4. Molecular Biomarkers, Therapeutic Approaches, and Neuroinflammation

During the past decade, the scientific community has struggled to find appropriate biomarkers in order to identify people at risk and patients in preclinical stages of Parkinson’s disease. Unfortunately, PD is a complex syndrome with different clinical subtypes and a wide variability in disease course in the need of diverse biomarkers, clinical, genetic, biochemical, and imaging [88]. Many promising biomarkers have been proposed so far from the search concerning neuroinflammation and PD. Polymorphisms in genes such as *LRRK2*, *S100B*, and *NURR1*, associated with inflammation, were found to increase the risk for PD [89,90]. Consequently, this could be translated into biomarkers for prognosis or/and diagnosis of PD, measuring the expression levels of their inflammatory proteins in CSF.

Ubiquitin C-terminal hydrolase-L1 (UCH-L1) is a deubiquitinating enzyme that helps regulate the metabolism of other brain proteins, mainly by removing excessive, oxidized, or misfolded proteins expressed in neurons [91]. Therefore, UCH-L1 prevents the aggregation of intracellular Lewy bodies, while its impairment or malfunction results in the reduced degradation of a-synuclein. A polymorphism (S18Y) found in its gene has been associated with protection from sporadic Parkinson’s disease, offering a specific antioxidant protective function [92]. Moreover, studies have demonstrated a substantial decrease of UCH-L1 concentrations in the CSF of PD patients compared to normal controls and patients suffering from other parkinsonian syndromes [93]. Specifically, PD patients had the lowest levels of CSF UCH-L1, a result that could function as a diagnostic marker for PD. To strengthen the specificity of the biomarker, the CSF a-synuclein levels could also be measured as there is a highly positive correlation between these two proteins.

β-Glucocerebrosidase (GCase) is a lysosomal hydrolase, encoded by the *GBA1* gene that plays a major role in α-synuclein degradation [94]. Loss-of-function mutations in *GCase* result in a rare, autosomal recessive lysosomal storage disorder called Gaucher disease (GD) [95], which is a clinical association that revealed the link between GD and parkinsonism [96]. Specifically, it was noted that a subset of Gaucher disease patients developed parkinsonian symptoms and that the prevalence of PD was higher among relatives of GD patients [97]. The mechanism that leads to the pathogenesis of synucleinopathies correlates with the loss of function mutations in the *GCase* gene (*GBA1*) and the alterations in sphingolipid metabolism. The functional loss of GCase affects lysosomal protein degradation, increases a-synuclein levels in neurons, and results in neurotoxicity through aggregation dependent mechanisms [98]. Both lysosomal dysfunction and α-synuclein aggregation seem to contribute to the pathogenesis of PD. Studies indicated that the combined determination of oligomeric a-synuclein/total a-synuclein and β-Glucocerebrosidase activity could improve the accuracy of PD diagnosis and further highlight the need for multiple different biomarkers in order to succeed in the early detection of PD patients [99].

Another molecular biomarker that has proven to be important in detecting neuroinflammation and to have diagnostic value for Parkinson’s disease is CCL28 (Mucosae-associated epithelial chemokine; MEC). CCL28 is a chemokine constitutively expressed in mucosal tissue and is moderately expressed in the small intestine, kidney and brain: in neurons rather than glia cells [100,101]. It seems to bridge the innate and adaptative immune response; the C-terminus has antimicrobial activity, and the N-terminus mediates lymphocyte migration. CCL28 was the only biomarker that was upregulated in PD patients compared to controls in a recent study [102]. The elevated levels in CSF might correspond to the idea that viral and microbial infections as well as altered gut-microbiota increase the risk of PD or that they may even be an early trigger of the disease. Another possible reason of elevated levels of CCL28 in CSF might be its release from degenerating neurons.

Plasminogen activators (PAs) are a class of proteolytic enzymes best-known for their anti-clotting properties and have been recently studied as possible inflammatory biomarkers in Parkinson’s disease [103]. PAs include tissue and urokinase PAs (tPA and uPA) as well as the endogenous inhibitor, PA inhibitor-1 (PAI-1). Pas seem to play a pivotal role in axonal regeneration. They also regulate extracellular matrix (ECM) remodeling, fibrinolysis, neuronal cell migration, and neuronal plasticity. More importantly, PAs released from neurons are required to prime pro-inflammatory cytokine production by microglia [104,105]. Then, microglia can also secrete their own Pas [106]. Therefore, plasminogen activator expression could serve as a novel and reliable biomarker for CNS inflammation as indicated by a recent study [106].

Mitochondrial dysfunction has been proven to induce neuroinflammation and neurodegeneration and vice versa. FGF21 (Fibroblast growth factor 21), besides being involved in a plethora of metabolic pathways [107], has recently been related to dysfunctional mitochondrial processes in neurons. When mitochondrial dynamics become impaired, neurons activate a multibranched stress response that culminates in the release of FGF21. The induction of neuron derived FGF21 has also been detected in the brains of mouse models of tauopathy and prion disease. Hence, FGF21 has been attributed a role as a mitokine and has been proposed as a candidate marker of brain mitochondrial dysfunction [108].

While the impact of neuroinflammation in the pathogenesis and the course of Parkinson’s disease is non disputable, the main concern remains the possible therapeutic tools that arise from this extensive research.

First, experimental evidence and animal studies showed a favorable result toward non-steroidal anti-inflammatory drugs, specifically ibuprofen and piroxicam, that seemed to reduce the risk for Parkinson’s disease [109,110,111]. However, epidemiological studies and meta-analysis did not confirm the positive effect of NSAID in either reducing the risk of PD, or modifying the disease course [112,113].

Other potential immunomodulatory therapies based on in vitro studies are anti-TNF therapies. TNF has been found to cause severe damage in dopaminergic neurons in vitro and the use of nonspecific TNF inhibitors, for example, thalidomide, had positive results in some MPTP mice and LPS rat model experiments [114,115]. Additionally, an epidemiological study revealed a lower incidence of PD in patients with inflammatory bowel disease under anti-TNF treatment in comparison with patients with no exposure to this specific treatment [116].

Scientists working on Isobavachalcone, a main component of a Chinese herb medicine Psoralea corylifolia in mouse models found that it could act as a neuroprotective and immunomodulatory factor. Ιsobavachalcone seems to act by blocking NF-κΒ signaling, resulting in improving motor deficits, alleviating neuronal necrosis, and at the same time decreasing the expressions of ΙL-6 and IL-1 [117].

Recently, in the field of immunomodulatory treatments, several immunotherapies targeting a-synuclein have emerged in an attempt to remove a-synuclein from the extracellular space and consequently reduce its aggregates in the brain. Similar clinical vaccination trials are conducted in Alzheimer’s disease, targeting amyloid β, and more recently intracellular tau protein [118]. Immunotherapeutic modalities targeting a-synuclein have steadily developed with several active and passive immunotherapies [119]. The active immunization derives from antibodies against a-synuclein that were generated in an animal immune system. The first designed vaccine was able to generate high titers of antibodies against aggregated a-synuclein, and the immunization succeeded in decreasing the deposition of a-synuclein and the striatal degeneration [120]. In the passive immunization, the antibodies administered are against different domains of a-synuclein [121,122]. The aim is the stimulation of microglia via these specific antibodies, the clearance of the extracellular a-synuclein, and prevention of cell-to-cell transfer of a-synuclein.

## 5. Conclusions

Neuroinflammation is a fundamental immune response to protect neurons from harm and compensate for neuronal damage, but at the same time, its neurotoxic effects exacerbate neuron damage. Furthermore, neuroinflammatory response is regulated by immune cells such as microglia, astrocytes, and peripheral immune cells, and by cytokines and chemokines. In this review, we focused on Parkinson’s disease and showed that dopaminergic neuronal loss is accompanied by inflammatory changes in microglia, astrocytes, innate immune cells, and infiltrating peripheral immune cells, and we tried to highlight the cause and effect relationship.

The main aim of the study of the pathogenesis of Parkinson’s disease is the development of new therapeutic approaches. The available therapies only treat the symptoms of the disease without stopping or slowing the neurodegenerative process. Neuroinflammation has gained an important role over time in the pathogenesis of Parkinson’s disease. Several treatments, targeting this process at different levels, have been experimented, have become an attractive option, and warrant further investigation. In most cases they have been proven successful when tested in animal models, but when going into clinical trials, the results are rather disappointing.

The more thoroughly we study the role of the immune system in Parkinson’s disease, the better the chances of discovering an effective immunomodulatory treatment.
